# Cardiac thin filament regulation and the Frank–Starling mechanism

**DOI:** 10.1007/s12576-014-0314-y

**Published:** 2014-05-01

**Authors:** Fuyu Kobirumaki-Shimozawa, Takahiro Inoue, Seine A. Shintani, Kotaro Oyama, Takako Terui, Susumu Minamisawa, Shin’ichi Ishiwata, Norio Fukuda

**Affiliations:** 1 Department of Cell Physiology, The Jikei University School of Medicine, 3-25-8 Nishi-shinbashi, Minato-ku, Tokyo, 105-8461 Japan; 2 Department of Cardiac Surgery, The Jikei University School of Medicine, Tokyo, Japan; 3 Department of Physics, Faculty of Science and Engineering, Waseda University, 3-14-9 Okubo, Shinjuku-ku, Tokyo, 169-0072 Japan; 4 Department of Anesthesiology, The Jikei University School of Medicine, Tokyo, Japan; 5 Waseda Bioscience Research Institute in Singapore (WABIOS), 11 Biopolis Way, #05-01/02 Helios, Singapore, 138667 Singapore

**Keywords:** Calcium, Cardiac muscle, Troponin, Titin

## Abstract

The heart has an intrinsic ability to increase systolic force in response to a rise in ventricular filling (the Frank–Starling law of the heart). It is widely accepted that the length dependence of myocardial activation underlies the Frank–Starling law of the heart. Recent advances in muscle physiology have enabled the identification of the factors involved in length-dependent activation, viz., titin (connectin)-based interfilament lattice spacing reduction and thin filament “on–off” regulation, with the former triggering length-dependent activation and the latter determining the number of myosin molecules recruited to thin filaments. Patients with a failing heart have demonstrated reduced exercise tolerance at least in part via depression of the Frank–Starling mechanism. Recent studies revealed that various mutations occur in the thin filament regulatory proteins, such as troponin, in the ventricular muscle of failing hearts, which consequently alter the Frank–Starling mechanism. In this article, we review the molecular mechanisms of length-dependent activation, and the influence of troponin mutations on the phenomenon.

## Introduction

**Fig. 1:**
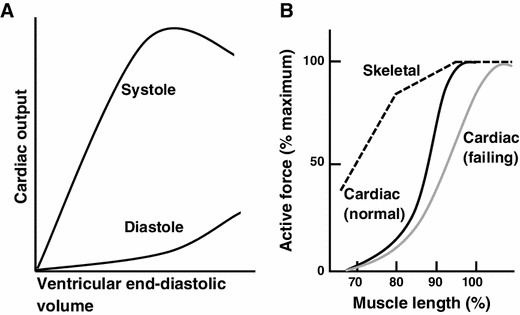
**a** Schematic illustration of the relationship between end-diastolic volume and peak systolic pressure during ventricular contraction in vivo [cf. 1]. **b** Schematic illustration showing the length dependence of activation in cardiac (*solid black line*) and skeletal (*dashed black line*) muscles. Muscle length is normalized with respect to the length at which active force is maximal for each type of muscle. The length dependence of activation is more pronounced in cardiac muscle than in skeletal muscle in the physiological length range (i.e., 80–100 %) [e.g., 40]. It has been reported that the slope of the length dependency is less steep in the myocardium of failing hearts (*solid gray line*) [e.g., 67, 68]

More than a century ago, Otto Frank in Germany and Earnest Starling in England discovered a fundamental principle in cardiovascular physiology, that an increase in ventricular filling enhances the systolic performance of the heart (i.e., the Frank–Starling law of the heart; see Fig. 1, [1]). Frank discovered the law in isolated perfused frog hearts, and Starling, in experiments with anesthetized dogs, verified that the Law operates in vivo on a beat-to-beat basis. In the 1980s, the newly developed non-invasive technologies revealed that the law is indeed utilized in healthy humans of various ages in a wide range of exercise levels [2, 3].

Both Frank and Starling understood that the law is the manifestation of the length dependence of active force development in skeletal muscle tissues; it was originally discovered by Schwann in 1832 and then became well known to physiologists during the second half of the nineteenth century ([4]; Fig. 1). The phenomenon seen in cardiac muscle is termed “length-dependent activation”, and it has been widely employed to analyze the Frank–Starling law of the heart.

It is generally known that the law operates not only in healthy conditions but also in diseased conditions, and the slope of the relationship of ventricular volume versus cardiac output becomes blunted as the malignity progresses. Our recent studies revealed that the attenuation of length-dependent activation becomes prominent under conditions where myocardial active force production capacity is either increased or decreased [5–8] (see discussions below).

In this article, we summarize the molecular mechanisms of length-dependent activation, focusing on the roles of titin (connectin) and thin filament regulatory proteins, and the maladaptive responses to disease-induced changes in the thin filament “on–off” regulation.

## General scheme of cardiac excitation–contraction coupling

**Fig. 2:**
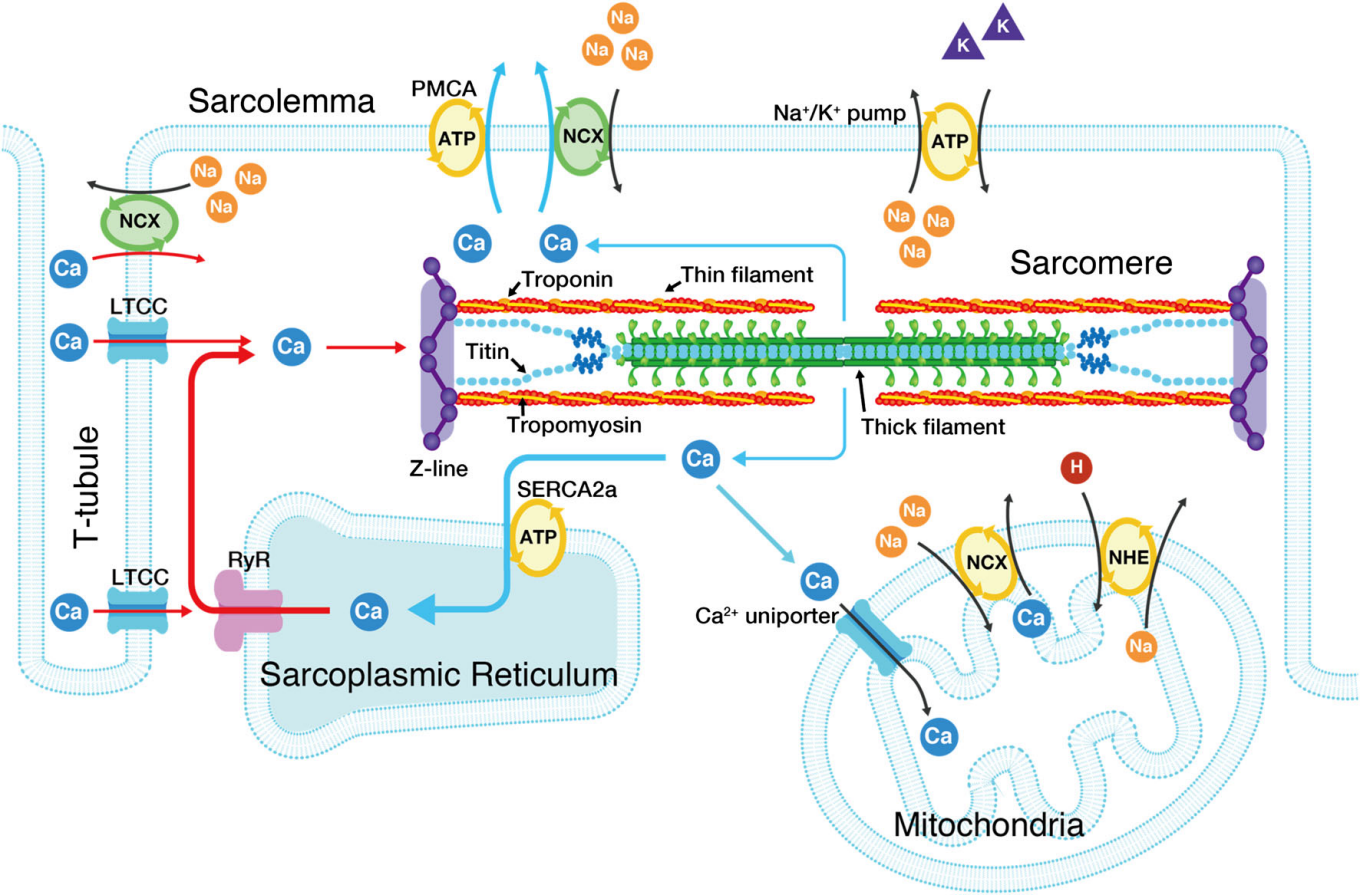
Schematic illustration indicating the structure of a cardiac sarcomere associated with the T-tubules. The influx of Ca^2+^ from the interstitial fluid during excitation causes the release of Ca^2+^ from the sarcoplasmic reticulum (SR). The released Ca^2+^ binds to troponin on the thin filaments and triggers sarcomeric contraction (systole) (see Fig. 3 for details). Relaxation (diastole) occurs as a result of uptake of Ca^2+^ by the SR Ca^2+^ pump, by extrusion of intracellular Ca^2+^ by Na^+^–Ca^2+^ exchangers, and partially by the sarcolemmal Ca^2+^ pump. Although the importance of mitochondria in pathophysiology has become increasingly evident, it remains unclear whether these organelles play a significant role in Ca^2+^ handling under physiological settings [e.g., 9]. The T-tubules and Z-lines run in parallel in cardiac muscle, causing Ca^2+^ sparks at/near the Z-lines [12, 13]. The thick and thin filaments, and titin are shown in this illustration (for simplicity, only two titin molecules per half thick filament are shown). Troponin and tropomyosin exist in the thin filaments, regulating actomyosin interaction in a [Ca^2+^]_i_-dependent manner. As described in detail in previous papers [e.g., 48–51], I-band titin is in a contracted state at the slack SL; straightening of the tandem Ig segment and then extension of the PEVK and N2B segments are thought to occur (resulting in passive force generation) in response to stretch. *LTCC* L-type Ca^2**+**^ channel, *RyR* ryanodine receptor, *PMCA* plasma membrane Ca^2+^ ATPase, *NCX* Na^+^–Ca^2+^ exchanger, *NHE* sodium**–**hydrogen exchanger

First, we briefly summarize the widely recognized perception of the molecular mechanisms of myocardial contraction and relaxation. In cardiac muscle, contraction is regulated by micromolar concentrations of intracellular Ca^2+^ on a graded basis [e.g., 9–11]. When the cellular membrane is depolarized upon entry of Na^+^ into the myocyte, Ca^2+^ enters the myocyte via sarcolemmal L-type Ca^2+^ channels (localized in the T-tubules) (Fig. 2). Because of the limited content, the Ca^2+^ entering the myocyte via the L-type Ca^2+^ channels does not directly activate myofilaments. Instead, it has an important role to trigger the release of Ca^2+^ from the sarcoplasmic reticulum (SR) via the Ca^2+^-induced Ca^2+^ release mechanism, resulting in the binding of Ca^2+^ to troponin C (TnC) and subsequent recruitment of myosin molecules to thin filaments (systole). While the Ca^2+^ release channels exist at the A-I junction in skeletal muscle, they run parallel with the Z-lines in cardiac muscle [e.g., 9, 12, 13].

In cardiac muscle, myofilaments are not fully activated even at the peak of systole, at least under physiologic conditions, because the intracellular Ca^2+^ concentration ([Ca^2+^]_i_) is maintained relatively low (up to ~10^−6^ M) [9]. Because of this low activation (or partial activation) property, cardiac myofilaments exhibit non-linear properties, such as length-dependent activation and spontaneous sarcomeric oscillations (SPOC) (see below).

[Ca^2+^]_i_ starts to fall prior to myocardial relaxation, and the following four systems are involved [9]: (1) sequestration into the SR by the Ca^2+^-ATPase pump (i.e., SERCA2a protein), (2) efflux via the sarcolemmal Na^+^/Ca^2+^ exchanger, (3) extrusion by the sarcolemmal Ca^2+^-ATPase pump, and (4) uptake into mitochondria via the Ca^2+^ uniporter. It is generally perceived that under physiologic conditions, (1) and (2) are primarily responsible for Ca^2+^ extrusion from the myocyte. Once [Ca^2+^]_i_ is lowered in the myocyte, Ca^2+^ dissociates from TnC, resulting in detachment of myosin molecules from thin filaments. Thereby, relaxation (diastole) occurs.

## Regulation of the “on–off” switching of cardiac sarcomeres: roles of troponin and tropomyosin

**Fig. 3:**
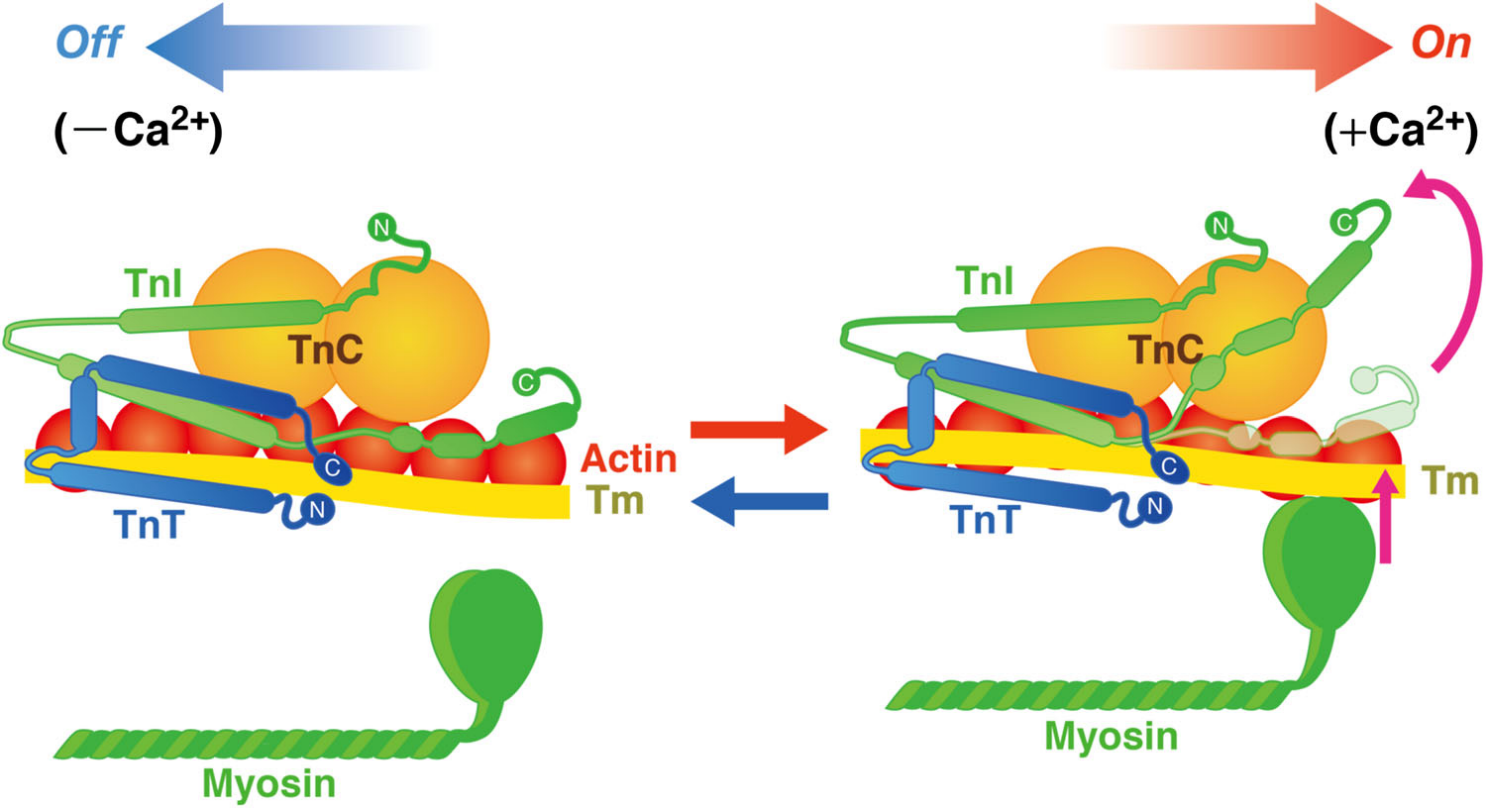
Structure and arrangement of cardiac thin filament proteins in the absence (off) and presence (on) of Ca^2+^. Shapes of the proteins are drawn primarily based on evidence from the work by Takeda et al. [15]. Upon Ca^2+^ binding to TnC, the C-terminus region of TnI dissociates from actin, allowing for Tm movement and, consequently, myosin binding to actin. *Arrows* indicate relative movements of Tm position in diastole and systole. *C* COOH terminus, *N* NH_2_ terminus. The equilibrium between the “off” state and the “on” state is a function of [Ca^2+^]_i_

In both cardiac and skeletal muscles, the state of the sarcomere is regulated by the troponin (Tn)–tropomyosin (Tm) complex on the thin filament, depending on [Ca^2+^]_i_. Tn is a heterotrimer of the distinct gene products: i.e., TnC, TnI, and TnT [e.g., 6, 14–16]. Two metal binding sites are present in the C-terminal domain of TnC that bind both Mg^2+^ and Ca^2+^ with relatively high affinity. However, because Mg^2+^ exists at relatively high concentrations inside cardiomyocytes (~1 mM compared with ~0.1 to ~1.0 μM for Ca^2+^), these sites are normally occupied by Mg^2+^. While fast skeletal TnC has two regulatory Ca^2+^-binding sites with relatively low affinity in the N-terminal domain of TnC, cardiac TnC has only one Ca^2+^-binding site, resulting in lower cooperativity of the “on–off” regulation of thin filaments. When [Ca^2+^]_i_ increases during systole, Ca^2+^ binds to the regulatory Ca^2+^-binding site, resulting in the onset of the conformational change of the thin filament (Fig. 3). During diastole, the C-terminal domain of TnI tightly binds to actin, and tropomyosin blocks the actomyosin interaction (“off” state). However, when Ca^2+^ binds to the regulatory Ca^2+^-binding site of TnC during systole, the C-terminal domain of TnI is dissociated from actin, and binds to the N-terminal domain of TnC, due to the enhanced TnC–TnI interaction (“on” state). It is generally considered that the transition from the “off” to “on” state is associated with a movement of tropomyosin on the thin filament [e.g., 6, 14–16].

The “on–off” equilibrium depends on the isoform of Tn subunits [e.g., 6, 14–16], as well as on the number of the strongly bound cross-bridges (e.g., rigor and ADP-bound cross-bridges; [17–19]). Likewise, the “on–off” equilibrium of the thin filament is modulated by phosphorylation/dephosphorylation by protein kinase A (PKA) or protein kinase C (PKC) [e.g., 14]. While PKA acts under physiologic conditions to control the heart’s inotropic and chronotropic states, various PKC isoforms are activated in diseased states [e.g., 14]. Both PKA and PKC decrease cardiac active force production (i.e., Ca^2+^ sensitivity), as a result of phosphorylation of various amino acid residues in TnI and TnT, with a common mechanism of the shift of the “on–off” equilibrium of the thin filament state toward the “off” state [e.g., 14].

It is worthwhile noting here that the “on–off” equilibrium of the thin filament state is also regulated thermally in a [Ca^2+^]_i_-dependent manner. Ishiwata [20] demonstrated in vitro that the Tn-Tm complex partially dissociates from F-actin in a reversible fashion (at >~39 °C), shifting the “on–off” equilibrium of the thin filament state toward the “on” state. Consistent with this notion, we demonstrated recently that cardiomyocytes isolated from adult rats exhibited contractions in response to an increase in the temperature (Δ*T* = 5 °C) of the bathing solution (36 °C) by means of illumination with an infra-red laser [21]. It should be stressed that this contraction does not accompany a change in [Ca^2+^]_i_ (cf. rapid cooling contracture; see [22]), hence an entirely novel sarcomere (thin filament)-based contraction. Further studies need to be conducted to investigate how the thin filament structure is altered upon heating-induced contractions.

## Spontaneous sarcomeric oscillations (SPOC): the third state of cardiac muscle

While it is widely believed that cardiac thin filaments take two states, either “on” (systole) or “off” (diastole), we have reported that cardiac sarcomeres exhibit a third state between contraction and relaxation [e.g., 23]. Namely, cardiac sarcomeres spontaneously oscillate showing a saw-tooth waveform at low Ca^2+^ concentrations, with a pCa (=−log [Ca^2+^]) of ~6.0 (Ca-SPOC) [e.g., 23]. This phenomenon was first reported by Fabiato and Fabiato [24] in 1978 and confirmed by other researchers, including us [25–28]. Likewise, cardiac sarcomeres exhibit a similar type of auto-oscillations in the presence of ADP and inorganic phosphate (Pi) with no Ca^2+^ (ADP-SPOC) [e.g., 23]. ADP-SPOC exhibits a waveform similar to that of Ca-SPOC, but with a lower (longer) oscillation frequency (period). In order to explore the relationship between Ca-SPOC and ADP-SPOC regarding ionic conditions, we constructed a three-dimensional state diagram showing contraction, relaxation or oscillation (SPOC) [26]. Our analysis revealed that the Ca-SPOC region (on the Ca^2+^ axis) and the ADP-SPOC region (on the ADP-Pi plane) are merged into a single SPOC region, sandwiched between the regions of contraction and relaxation [26]. Therefore, the three-dimensional diagram indicates that despite distinct ionic conditions, the molecular mechanism is common to both ADP-SPOC and Ca-SPOC, as mathematically shown in our recent work [29].

Interestingly, the frequency of SPOC (either Ca-SPOC or ADP-SPOC) is significantly correlated with the resting heart rate in various animal species [28, 30]. While the physiological role of sarcomeric auto-oscillations is still controversial, the findings of our previous studies [28, 30–32] and those of others [33] suggest that SPOC may play a role in efficiently promoting relaxation to neighboring sarcomeres. Clearly, future studies need to be conducted to clarify whether and how SPOC operates under physiological conditions.

## Early discoveries on length-dependent activation: [Ca^2+^]_i_ versus sarcomeres

Two articles were published in 1982 on the effect of “muscle length” in cardiac contraction. First, in a pioneering study using the Ca^2+^-sensitive photoprotein aequorin, Allen and Kurihara [34] simultaneously measured [Ca^2+^]_i_ and twitch force in living cardiac muscle (ferret ventricular muscle). This historically important study revealed that the length dependence of cardiac muscle activation is composed of two phases: i.e., a rapid, first phase that occurs independent of [Ca^2+^]_i_ based on sarcomeric activation, and a second phase that is coupled with the rise of [Ca^2+^]_i_ via enhanced Ca^2+^ release from the SR. Provided that the Frank–Starling mechanism operates on a beat-to-beat basis [1], it is reasonable to consider that the rapid first phase underlies length-dependent activation, at least under physiologic conditions.

**Fig. 4:**
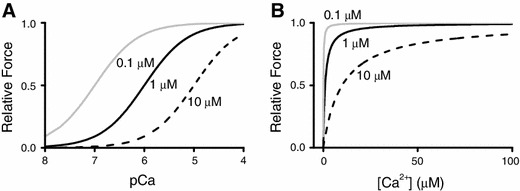
Active force plotted against pCa or [Ca^2+^]_i_. **a** Active force–pCa curves simulated based on the equation $F = \frac{1}{{1 + 10^{{h{\kern 1pt} \left( {{\text{pCa}} - {\text{pCa}}_{50} } \right)}} }}$ (i.e., $y = \frac{1}{{1 + 10^{{h{\kern 1pt} (x - pK_{\text{d}} )}} }}$), where *F*, pCa_50_ and *h* indicate relative force, the mid-point of each curve and the Hill coefficient, respectively (*x* pCa; *y* relative force). Three different apparent *pK*_d_ values for Ca^2+^-binding to TnC (calculated using hypothetical SL changes) were used for simulation, i.e., 0.1, 1 ,and 10 μM, at *h* = 1. **b** Active force–[Ca^2+^] curves simulated based on the equation $F = \frac{{\left[ {{\text{Ca}}^{2 + } } \right]^{h} }}{{{\text{EC}}_{50}^{h} + \left[ {{\text{Ca}}^{2 + } } \right]^{h} }}$ (i.e., $y = \frac{1}{{1 + \left( {\frac{{K_{\text{d}} }}{x}} \right)^{h} }}$), where EC_50_ indicates the mid-point of each curve (*x* [Ca^2+^]; *y* relative force). Three different apparent *K*_d_ values for Ca^2+^-binding to TnC (calculated using hypothetical SL changes) were used for simulation, i.e., 0.1, 1, and 10 μM, at *h* = 1. As shown in the graphs, Ca^2+^ sensitivity is more clearly indicated in (**a**) using pCa values (see text for details)

Another set of findings of direct evidence that myofibrillar contractility is increased with length was provided by Hibberd and Jewell [35] in experiments with chemically “skinned” myocardial preparations. Those authors observed that submaximal active force increases with an increase in sarcomere length (SL), resulting in the leftward shift of the force–pCa curve (hence, an apparent increase in Ca^2+^-binding to TnC associated with an increase in SL) (Fig. 4). It is important to note here that the mid-point of the force–pCa curve represents the apparent dissociation constant (*K*_d_) for Ca^2+^-binding to TnC (or myofibrils) in myocardial preparations. For example, when the pCa_50_ value is 6.0, then the apparent *K*_d_ of Ca^2+^-binding to TnC in the muscle preparation is 10^−6.0^ M (1 μM). Therefore, a SL-dependent increase in the pCa_50_ value indicates that the apparent *K*_d_ decreases via enhancement of the apparent affinity of TnC (or myofibrils) for Ca^2+^ (as discussed in [8]). Since the 1982 publication of the work by Hibberd and Jewel, the magnitude of length-dependent activation has been widely expressed by using ΔpCa_50_. In a hypothetical condition, if *K*_d_ decreases upon SL elongation from 10^−6^ to 10^−7^ M, the ΔpCa_50_ value, i.e., 1.0, represents the *K*_d_ value decreasing by an order of magnitude after SL elongation (Fig. 4, left). Here, one may point out that the degree of length-dependent activation can be more accurately represented by the difference between the absolute Ca^2+^ concentration ([Ca^2+^]) at half-maximal activation before and after SL elongation, i.e., ΔEC_50_, instead of ΔpCa_50_. However, if ΔEC_50_ is used, the position as well as the shape of the curve is impacted differently by the same magnitude of an increase or a decrease in *K*_d_ (Fig. 4, right), making it difficult to visualize the effect of lengthening. We therefore consider that, as was established by Hibberd and Jewell [35], and recently pointed out by Walker et al. [36], length-dependent activation should be expressed by ΔpCa_50_, based on the concept of *K*_d_ for Ca^2+^-binding to TnC (or myofibrils) in myocardial preparations.

## Sarcomeric conformational changes and length-dependent activation

In 1966, Gordon, Huxley and Julian reported that active force increases linearly as the overlap between the thick and thin filaments increases in the sarcomere of frog skeletal muscle [37]. They demonstrated that active force production of sarcomeres becomes less pronounced at stretched (>~2.2 μm) as well as at shortened (<~2.0 μm) states; the former coupled with a decrease in the thick and thin filament overlap and the latter with an increase in the thin filament double overlap (i.e., two thin filaments per cross-bridge). This theory fits well with the experimental findings on skeletal muscle [e.g., 38], but it can only marginally account for cardiac length-dependent activation (as discussed in [39]). For instance, both maximal and submaximal forces markedly increase upon an increase in SL from 2.0 to 2.2 μm in cardiac muscle [e.g., 40, 41], but the change in the thick–thin filament overlap is very small, given the length of the A-band and that of the I-band are ~1.6 and ~1.0 μm, respectively. Instead, it has been proposed that SL-dependent increases in active force result from an increase in the number of cross-bridges coupled with a reduction in interfilament lattice spacing (i.e., the spacing between the thick and thin filaments) [e.g., 19, 42–44]. The actomyosin interaction is a stochastic process, based on thermal fluctuation of actomyosin molecules [e.g., 45, 46]. It is therefore considered that the attachment of myosin heads to the thin filament is enhanced as the distance between the thick and thin filament is reduced. Indeed, osmotic compression by application of large molecules (such as dextran; MW, 500,000) in the bathing solution reportedly reduces the lattice spacing and increases contractile force, coupled with enhanced cross-bridge formation [19, 42–44]. Likewise, synchrotron X-ray diffraction studies have provided direct evidence that the lattice spacing is reduced as SL increases within the physiological range (i.e., ~1.8 to ~2.4 μm) in various animal species [e.g., 47]. These findings are consistent with the notion that length-dependent activation is, at least partly, a result of the lattice spacing reduction rather than filament overlap changes.

## Titin’s role as a modulator of interfilament lattice spacing

As illustrated in Fig. 2, titin (connectin) exists in the sarcomere of striated muscle cells [e.g., 48–51]. Titin is the largest protein known to date (i.e., 3–4 MDa) and functions as a passive force generator in cardiac and skeletal muscles at the long SL range. It has likewise been reported that titin operates as a molecular scaffold during myofibrilogenesis in striated muscles [e.g., 49]. Indeed, we demonstrated in vivo that the sarcomere structure is markedly disordered under conditions where titin expression (degradation) is reduced (enhanced) upon atrophy in skeletal muscle [52]. Another critical role of titin in muscle physiology is that it stabilizes the sarcomere structure by positioning the thick filaments in the center of the sarcomere following contraction [e.g., 48–51]. Because of this nature, sarcomeres can repeat contraction and relaxation without deterioration of their structure.

Titin spans from the Z-line to the M-line in the half-sarcomere of striated muscle [e.g., 48–51]. Titin develops passive force when its spring element in the I-band region is stretched [e.g., 48–51]. Titin’s I-band region has a complex sequence with different extensible segments: the tandem immunoglobulin (Ig) segments (tandemly arranged Ig-like domains), the PEVK segment [rich in proline (P), glutamate (E), valine (V) and lysine (K)] and the segment with a unique amino acid sequence (called “N2A” or “N2B”). Its A-band portion consists of simple patterns of Ig-like and fibronectin type 3 (Fn3) repeats. In cardiac muscle, two types of titin exist, i.e., stiff N2B titin (containing the N2B segment) and compliant N2BA titin (containing both N2B and N2A segments). N2BA titin contains an additional middle Ig segment, the N2A segment, and the PEVK segment of variable lengths, hence producing lower passive force. N2B titin is preferably expressed in the ventricles of small animals, and N2BA titin is dominantly expressed in the atria of large animals [e.g., 48–51]. In the ventricle of large mammals, including humans, both N2B and N2BA isoforms are expressed at ~1:~1, under healthy conditions [e.g., 48–51].

**Fig. 5:**
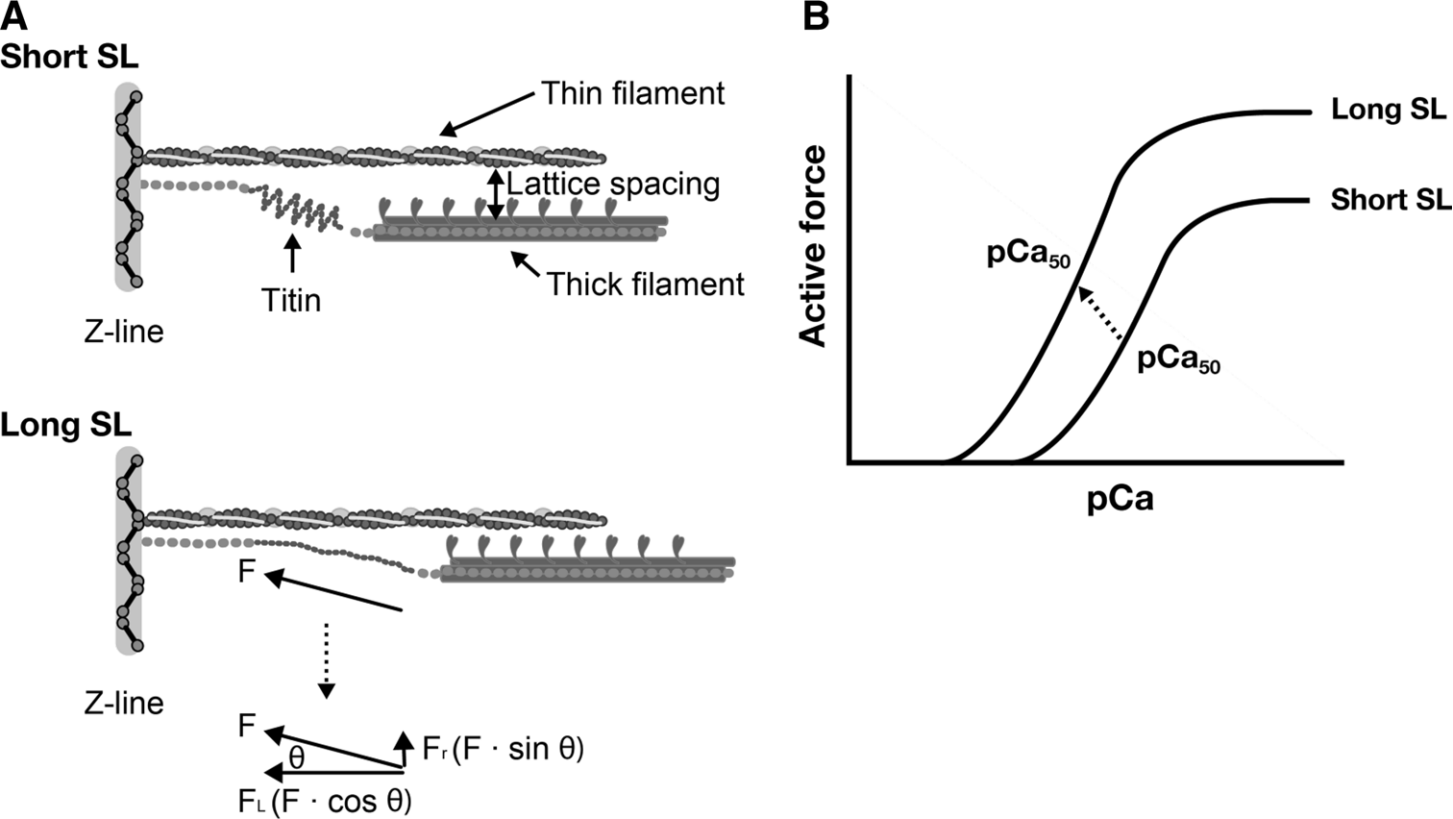
Schematic illustration showing the role of titin in the modulation of interfilament lattice spacing (**a**) and length-dependent changes in the force–pCa curve (**b**). At a long length, titin produces passive force (*F*) in the myofilament lattice. The force is divided into longitudinal (*F*_L_) and radial (*F*_R_) components, the latter of which reduces interfilament lattice spacing. The lattice reduction is likely to enhance myosin attachment to actin and, consequently, it causes a leftward shift of the force–pCa curve and increases the maximal Ca^2+^-activated force [see *arrow* (indicating the mid-point of the curve) in (**b**)]

In 2001, Cazorla et al. [53] measured force–pCa curves in skinned rat cardiomyocytes at different levels of passive force, and demonstrated that the magnitude of the SL-dependent increases in Ca^2+^ sensitivity (ΔpCa_50_) varies in response to passive force, rather than response to SL per se. Another important finding by Cazorla et al. is that the interfilament lattice spacing changes in response to a change in titin-based passive force. As shown in Fig. 5, titin binds to actin in/near the Z-line, and, therefore, the molecule produces lateral force as well as longitudinal force when stretched in the sarcomere [e.g., 48–51]. Cazorla et al. concluded that the lateral force pulls the thin filament closer to the thick filament, increasing the probability of myosin attaching to the thin filament. These series of findings by Cazorla et al. were followed by Fukuda et al. [54] who used cardiac muscles that express different titin isoforms, i.e., bovine ventricular and atrial muscles expressing N2B titin and N2BA titin at ~1:~1 and ~0.9:~0.1, respectively [54]. It was found that both length-dependent activation and length-dependent lattice reduction are clearly more pronounced in bovine left ventricle than in bovine left atrium, supporting the notion that titin regulates length-dependent activation by regulating the lattice spacing.

Later, the group of de Tombe challenged the lattice spacing hypothesis, providing evidence that the lattice spacing and Ca^2+^ sensitivity are not well correlated [55–57]. However, because the lattice spacing does not change linearly in response to a change in SL in striated muscle cells, Ca^2+^ sensitivity may not necessarily be closely correlated with the lattice spacing (as discussed in [8]).

Furthermore, it is likely that cross-bridge geometry (i.e., relative position of myosin molecules to the actin-binding sites on the thin filaments) is different in osmotic compression by high molecular weight polymers and lengthening. Therefore, the series of experimental findings by the de Tombe group [55–57] suggests that the lattice spacing does not solely determine the magnitude of length-dependent activation, and it does not rule out the involvement of the lattice spacing in the regulation of length-dependent activation.

Alternatively, it is possible that titin regulates length-dependent activation via thick filament structural change, as pointed out by us [41]. We applied limited trypsin treatment to rat ventricular trabeculae to selectively degrade titin (see [58] for the effects of limited trypsin treatment on sarcomere proteins) and found that the slope of the length–force curve became less steep. Since attenuation of the length-dependence was likewise observed under conditions where the lattice spacing (as indexed by muscle width) was not significantly changed, we concluded that a mechanism operating independent of the lattice spacing modulation was responsible. Because titin forms an integral component of the thick filament, tightly binding to myosin and myosin-binding protein C [59], our findings suggest that sarcomere extension by an external force may cause mechanical strain of the thick filament via titin’s longitudinal force, allowing myosin to attach to actin (i.e., active force production). Whether or not this mechanism operates under physiological conditions warrants future investigation, presumably by means of synchrotron X-ray diffraction studies.

## Thin filament-based regulation of length-dependent activation

To the knowledge of the authors, the group of Gulati was the first to report that the thin filament system is involved in the regulation of length-dependent activation [60, 61]. Namely, those authors reported that TnC plays a role as a “SL sensor” that changes its Ca^2+^-binding affinity as the magnitude of the overlap of the thick and thin filament varies. This unique proposal is based on the observation that the length-dependent shift of the force–pCa curve in skinned cardiac muscle is diminished after the exchange of TnC for the fast skeletal isoform that exhibits a lesser magnitude of length-dependent activation [60, 61]. Later, the group of Moss challenged this proposal providing two sets of evidence that (1) the length dependence of skeletal muscle activation is unaltered after TnC is exchanged for cardiac TnC [62], and (2) the overexpression of skeletal TnC in cardiac myocytes does not influence length-dependent activation [63], rendering the role of TnC as a “SL sensor” unlikely, at least under steady-state conditions. Nevertheless, it should be stressed that the feedback loop between Ca^2+^-binding to TnC and cross-bridge formation operates under conditions where the active force changes with time, such as in physiologic twitch contractions in intact cardiac preparations [64].

Approximately two decades ago, the group of Solaro successfully developed a mouse model expressing slow skeletal TnI (that lacks cardiac-specific protein kinase A phosphorylation sites; ssTnI), and this model has been widely used to investigate the physiological properties of TnI under various conditions [65]. Interestingly, ventricular preparations expressing ssTnI exhibits greater Ca^2+^ sensitivity of force than control muscle [65]. This is likely due to the acceleration of the transition from the “off” state to the “on” state of the thin filament. Those authors demonstrated that the length-dependent activation was less pronounced in ssTnI muscle preparations [66]. Similarly, we demonstrated by using Tn exchange techniques that the “on–off” equilibrium of the thin filament state plays a critical role in length-dependent activation: i.e., the replacement of endogenous Tn with exogenously added rabbit fast skeletal Tn attenuated the length dependency in skinned porcine ventricular muscle, to a magnitude similar to that observed in rabbit fast skeletal muscle (see also [5–7] for differing magnitudes of length-dependent activation in cardiac and skeletal muscles). Considering the findings that (1) Ca^2+^ sensitivity is increased and (2) cross-bridge kinetics is accelerated upon replacement with rabbit fast skeletal Tn [5], the switching of Tn from the cardiac to fast skeletal isoform likely accelerates the transition from the “off” state to the “on” state of the thin filament (similar to the finding in [66]). It is therefore considered that length-dependent activation is attenuated as a result of a decrease in the number of “recruitable” cross-bridges. We likewise demonstrated that an increase in titin-based passive force, induced by manipulating the pre-history of stretch (as in [5]), enhanced length-dependent activation in both control and fast skeletal Tn-reconstituted cardiac muscles, suggesting that this phenomenon is coupled with both titin-based passive force and “on–off” switching of the thin filament state, with titin-based passive force acting as a trigger in this phenomenon.

Therefore, the “on–off” equilibrium of the thin filament state, which is regulated primarily by Tn (and Tm) under physiologic conditions, is likely to determine the magnitude of length-dependent activation, in concert with titin-based interfilament lattice pacing regulation. We hereby conclude that, in myocardial preparations from healthy hearts, length-dependent activation is triggered by the structural change of the sarcomere, i.e., titin-based interfilament lattice spacing reduction, and the number of myosin attaching to actin is dependent on the “on–off” equilibrium of the thin filament state [5–8].

## Pathophysiology of the Frank–Starling relation: thin filament-based modulations

One of the clinically important aspects of the physiology of failing hearts is the depression of the Frank–Starling mechanism. Some authors reported compromised length-dependent activation in failing human hearts [67, 68]; however, others reported that this property functions nearly normally in humans with heart failure [69, 70]. Recent studies support a view that the Frank–Starling mechanism is depressed in pathological hearts [i.e., hypertrophic (HCM) and dilated (DCM) cardiomyopathies] caused by troponin mutations via different molecular mechanisms.

**Fig. 6:**
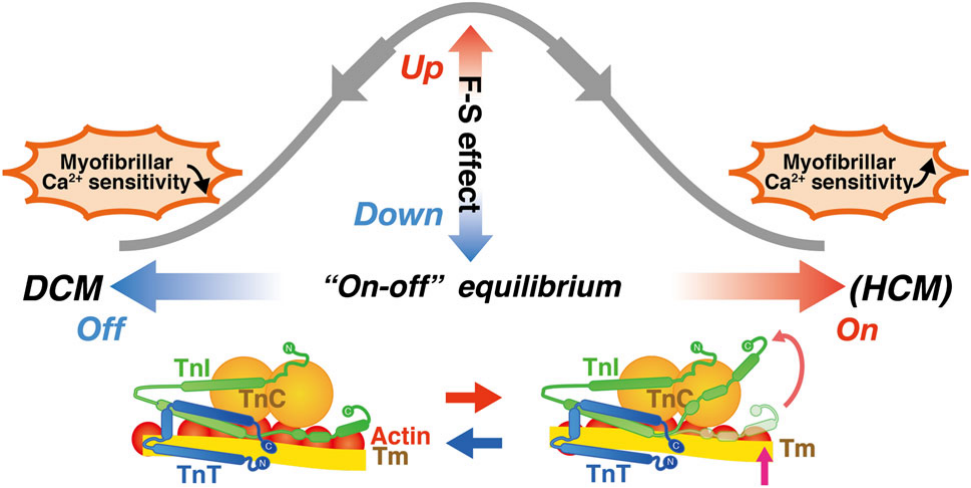
Relationship between the “on–off” equilibrium of the thin filament state and the magnitude of length-dependent activation. Length-dependent activation [noted as “*F*–*S* (Frank–Starling) effect”] becomes less pronounced when the equilibrium is shifted toward either the “on” state or the “off” state, but via different mechanisms. Namely, on the one hand, when the equilibrium is shifted toward the “on” state (as presumably in some mutated HCM hearts), the length dependence becomes attenuated, coupled presumably with a decrease in the number of recruitable (+ATP) cross-bridges (cf. [5–7]). On the other hand, when the equilibrium is shifted toward the “off” state (as in ΔK210 hearts; [8]), the length dependence becomes attenuated, via reduction in thin filament cooperative activation and the ensuing suppression of myosin attachment to actin upon lattice spacing reduction

Recent genetic analyses have revealed that inherited cardiomyopathies, i.e., hypertrophic (HCM), dilated (DCM), and restrictive (RCM) cardiomyopathies, are associated with mutations of genes for sarcomeric and cytoskeletal proteins, including cardiac troponin (see [16] and references therein). HCM is an autosomal dominant disorder of the heart that has characteristic symptoms of an asymmetric left ventricular hypertrophy and impaired diastolic function (see [16] and references therein). Genotyping studies have identified a disease-causing mutation in ~70 % of all patients with HCM (see [71] and references therein). Thick filament-encoding genes account for ~80 % of sarcomere mutations, and ~18 % of the mutations are located in thin filament-encoding genes, i.e., *TNNI3* (cTnI), *TNNT2* (cTnT), *TNNC1* (cTnC), *TPM1* (α-tropomyosin), and *ACTC1* (α-cardiac actin); the remaining ~2 % is attributable to *MYL3* and *MYL2* (encoding regulatory and essential myosin light chains) and *TTN* (encoding titin) (see [71] and references therein). To date, 27 mutations of TnT, 26 mutations of TnI, and 1 mutation of TnC have been associated with HCM (see [71] and references therein). In a recent extensive study using human ventricular samples, Sequeira et al. [71] reported that length-dependent activation is depressed, accompanied by an increase in Ca^2+^ sensitivity in ventricular samples from patients with HCM having mutations in genes encoding thick and thin filament proteins. They provided evidence by using the Tn reconstitution technique (as in [5, 7, 8]) that mutant troponin (K280N and R145W) impairs length-dependent activation. Their biochemical studies likewise indicate reduced PKA-based phosphorylation of sarcomere proteins (i.e., TnI and MyBP-C), which may in part account for the observed depressions in length-dependent activation (as first reported by us; see [72]). However, given the experimental evidence that length-dependent activation is still less in diseased preparations after normalized phosphorylation with PKA, the depression in the length dependency is likely the result of posttranslational modifications associated with a shift of the “on–off” equilibrium of the thin filament state, presumably toward the “on” state (as judged from increased Ca^2+^ sensitivity in HCM preparations; [71]), other than PKA phosphorylation or altered protein–protein interactions. These findings can be interpreted in line with those of our previous studies [5–7]: i.e., length-dependent activation becomes less pronounced under conditions where Ca^2+^ sensitivity is increased, coupled with a decrease in the number of “recruitable” cross-bridges (Fig. 6).

DCM is characterized by the enlargement of cardiac chambers and systolic/diastolic dysfunctions (see [16] and references therein). Because of a poor prognosis and malignancy of the heart’s pump functions, patients with DCM account for as high as ~50 % of those who are destined for a heart transplant [73–75]. DCM is caused by mutations in genes for various proteins in cardiomyocytes, including sarcomere proteins [76]. Familial DCM constitutes ~30 % of all non-ischemic DCM, and various classes of DCM-causing mutations in thin filament regulatory proteins have recently been identified [16, 76]. Initial genetic analyses demonstrated that genes for cytoskeletal proteins are involved in DCM, but subsequent studies revealed that mutations of cardiac sarcomeric proteins, such as TnT, TnI, TnC, actin, myosin-binding protein C, α-tropomyosin, and β-myosin heavy chain, are also involved in DCM (see [16] and references therein). Six mutations in TnT, 1 mutation in TnI, and 1 mutation in TnC have been reported (see [16] and references therein). Deletion mutation ΔK210 in cardiac TnT (cTnT) is a recurrent DCM-causing mutation identified worldwide [e.g., 77, 78]. In a recent study, the group of Morimoto created a knock-in mouse model with a DCM-causing deletion mutation ΔK210, and demonstrated that the mutation causes a marked depression in Ca^2+^ sensitivity [78]. By using the ventricular preparations from ΔK210 mice, we recently reported that length-dependent activation is markedly depressed [8]. Namely, an increase in SL from 1.90 to 2.20 μm shifted the mid-point (pCa_50_) of the force–pCa curve leftward by ~0.21 pCa units in wild-type (WT) preparations. In contrast, in ΔK210 muscles, Ca^2+^ sensitivity was lower by ~0.37 pCa units, and the SL-dependent shift of pCa_50_ was less pronounced (~0.10 pCa units), regardless of PKA treatment [8]. The rate of active force redevelopment was lower in ΔK210 preparations than in WT preparations, showing blunted thin filament cooperative activation, and an increase in the number of strongly bound cross-bridges by MgADP increased ΔpCa_50_ to ~0.21 pCa units [8]. Therefore, these findings are consistent with the notion that (1) the magnitude of length-dependent activation is less pronounced when the “on–off” equilibrium of the thin filament state is either increased or decreased, and (2) this phenomenon becomes most pronounced under partial activation states (Fig. 6; as discussed in [8]).

As discussed above, titin isoform switching occurs in DCM. Namely, the expression ratio of N2BA titin to total titin (N2B titin + N2BA titin) reportedly increases in DCM [79, 80]. Although the increased PKC activity may increase titin-based passive force via phosphorylation of the PEVK segment [81], coupled with reduced PKA-based phosphorylation of the N2B segment [e.g., 49–51], it is reasonable to consider that the overall titin-based passive force decreases in DCM. Because titin regulates length-dependent activation via modulation of interfilament lattice spacing (Fig. 5), its isoform shift to the N2BA-dominant state is likely to attenuate length-dependent activation via reduction in its lateral force in the sarcomere. Likewise, an increase in PKC activity during progression of DCM may alter the Frank–Starling mechanism, independent of titin-based regulations. Montgomery et al. [82] reported that the Frank–Starling mechanism is depressed in mouse hearts overexpressing PKCε. PKCε phosphorylates troponin T and troponin I, thereby lowering myofilament activation by shifting the “on–off” equilibrium of the thin filament state toward the “off” state (as in [14]), resulting in the attenuation of length-dependent activation via reduced thin filament cooperative activation (as in ΔK210 hearts). Therefore, we conclude that length-dependent activation is depressed in DCM, and the mechanisms involve (1) reduced thin filament cooperative activation (and ensuing reduction of cross-bridge formation upon lattice reduction), and (2) titin’s isoform switching, i.e., from N2B isoform to N2BA isoform, and the resultant reduction of titin’s modulation of interfilament lattice spacing.

RCM is less common than HCM or DCM, characterized by impaired diastolic filling of the left ventricle and normal or near normal systolic function [16, 83]. Typically, RCM hearts exhibit normal or near normal ventricular wall thickness [16, 83]. Six mutations of human cardiac troponin I have been found to be associated with RCM [16, 83]. To the knowledge of the authors, no systematic studies have been conducted to investigate how the Frank–Starling mechanism may or may not be altered in RCM. Considering, however, the increased Ca^2+^ sensitivity, it is expected that length-dependent activation is attenuated because of the decrease in the number of “recruitable” cross-bridges associated with a shift of the equilibrium of the thin filament state toward the “on” state. However, systematic studies are needed to investigate whether and how length-dependent activation is altered in RCM.

To summarize, recent findings suggest that the Frank–Starling mechanism is depressed in HCM and DCM, but via different mechanisms, i.e., the former coupled with enhanced thin filament cooperative activation and the latter with reduced thin filament cooperative activation (and presumably, titin isoform switching from N2B isoform to more compliant N2BA isoform). Future studies are warranted to fully and systematically investigate how various mutations in thin filament regulatory proteins affect the Frank–Starling mechanism, in various types of cardiomyopathy.

## Conclusion

Length-dependent activation in ventricular preparations forms the basis for the Frank–Starling law of the heart and is the principle in cardiac physiology. Almost a century after the discovery of the law, recent advances in physiology technologies have successfully revealed the molecular mechanisms of length-dependent activation: i.e., titin plays as a triggering factor in this phenomenon by reducing interfilament lattice spacing via its lateral force in the sarcomere, and the magnitude of the cross-bridges formed upon titin extension depends on the number of “recruitable” cross-bridges. The “on–off” equilibrium of the thin filament state varies in HCM or DCM, thereby changing the magnitude of length-dependent activation. Future studies are warranted to systematically investigate the relationship between the “on–off” equilibrium of the thin filament state and the magnitude of length-dependent activation, in HCM, DCM and RCM.

## Abbreviations

[Ca^2+^]_i_: Intracellular Ca^2+^ concentration
SL: Sarcomere length
SPOC: Spontaneous sarcomeric oscillations
